# The Kidneys and Urine

**Published:** 1843-07-08

**Authors:** 


### BIBLIOGRAPHICAL NOTICES.

*The Kidneys and Urine.* By J. J. Berzelius. Translated from the German, by M. H. Boyé and F. Leaming, M. D.

This book is designed expressly for the use of those who wish to have a knowledge of the chemical qualities of the urine, and of the best methods of examining this secretion in various diseases. In regard to the history of each ingredient, it is more detailed, more complete than any thing that has yet been written, and its authority is such as few will venture to question.

It is an agreeable thing to see a man like Berzelius, in advanced life, with unimpaired faculties, still eagerly pursuing that science to which his whole life has been devoted. It is still more agreeable to witness the homage he receives from all chemists; he seems to be regarded as the *father* of modern chemistry. His "*Year-Book*" manifests his unconquerable energy, as well as the intense interest with which he watches the progress of his science. There you may see him judging the merits of every thing produced, and stamping every thing that is good and true with his own seal. Every fact, every process, every theory that is proposed by any chemist in the world, he examines, and discusses and settles.

The book before us is the first attempt to make known the wonderful excellence of this man to those who are acquainted with the English language only. It is a faithful translation of part of his *Animal Chemistry*, and is on a subject exciting at present a good deal of attention in England. We believe this interest was first awakened by Dr. Prout. Certainly, no one can read his elaborate work on the "Stomach," &c., without being convinced of the necessity of a chemical knowledge of the urine in many complaints. According to him the existence of cystic oxide is not so uncommon as was supposed; sugar is very frequently present; and oxalic acid often accompanies certain deteriorated conditions of the system which cannot be relieved before measures are taken to prevent the formation of this acid. These are facts of great importance, not to science merely, but to many a sufferer whose life is rendered miserable by the ignorance of his physician. Dr. Prout's work, however, treats only of the *diseases* dependent on or accompanied by deranged conditions of the urine. He prescribes no methods by which the healthy or deleterious substances may be detected. We believe Willis is the only author who has given such prescriptions, but after a careful examination we have concluded that some of them are useless, and many of them incomprehensible. It is exactly this deficiency in English science which this treatise of Berzelius completely meets. The tests he prescribes are so simple and easily applied, that any one in half an hour's time can ascertain the presence of all the ingredients, and that too by a very small outlay for chemical tests. Half a dozen test tubes, and a dozen bottles of tests, costing in all less than five dollars, will enable a physician to make any observations he may require, and have materials enough for daily observations for several years. We have been in the habit for some time past of using the means he has pointed out for the examinations, and we declare we would on no account be without them; the satisfaction of knowing positively that a patient has or has not kidney disease, is worth much trouble, without reference to the improved chances of benefit to the patient.

